# Fear Detection Using Electroencephalogram and Artificial Intelligence: A Systematic Review

**DOI:** 10.3390/brainsci15080815

**Published:** 2025-07-29

**Authors:** Bladimir Serna, Ricardo Salazar, Gustavo A. Alonso-Silverio, Rosario Baltazar, Elías Ventura-Molina, Antonio Alarcón-Paredes

**Affiliations:** 1 Centro de Innovación, Competitividad y Sostenibilidad, Universidad Autónoma de Guerrero, Acapulco 39640, Guerrero, Mexico; blad.sernav@gmail.com; 2 SECIHTI-Universidad Autonoma de Guerrero, Chilpancingo 39070, Guerrero, Mexico; rsalazarlo@secihti.mx; 3 Facultad de Ingeniería, Universidad Autónoma de Guerrero, Chilpancingo 39079, Guerrero, Mexico; gsilverio@uagro.mx; 4 División de Estudios de Posgrado, Tecnológico Nacional de México/Campus León, León 37290, Guanajuato, Mexico; charobalmx@yahoo.com.mx; 5 Centro de Innovación y Desarrollo Tecnológico en Computo, Instituto Politécnico Nacional, Mexico City 07738, Mexico; eventuram@ipn.mx; 6 Centro de Investigación en Computación, Instituto Politécnico Nacional, Mexico City 07738, Mexico

**Keywords:** electroencephalography (EEG), fear detection, artificial intelligence (AI), machine learning (ML), emotion recognition, brain–computer interface (BCI), neural signals, PRISMA, affective computing, EEG signal processing, brainwave analysis, affective computing, neurotechnology

## Abstract

**Background/Objectives:** Fear detection through EEG signals has gained increasing attention due to its applications in affective computing, mental health monitoring, and intelligent safety systems. This systematic review aimed to identify the most effective methods, algorithms, and configurations reported in the literature for detecting fear from EEG signals using artificial intelligence (AI). **Methods:** Following the PRISMA 2020 methodology, a structured search was conducted using the string (“fear detection” AND “artificial intelligence” OR “machine learning” AND NOT “fnirs OR mri OR ct OR pet OR image”). After applying inclusion and exclusion criteria, 11 relevant studies were selected. **Results:** The review examined key methodological aspects such as algorithms (e.g., SVM, CNN, Decision Trees), EEG devices (Emotiv, Biosemi), experimental paradigms (videos, interactive games), dominant brainwave bands (beta, gamma, alpha), and electrode placement. Non-linear models, particularly when combined with immersive stimulation, achieved the highest classification accuracy (up to 92%). Beta and gamma frequencies were consistently associated with fear states, while frontotemporal electrode positioning and proprietary datasets further enhanced model performance. **Conclusions:** EEG-based fear detection using AI demonstrates high potential and rapid growth, offering significant interdisciplinary applications in healthcare, safety systems, and affective computing.

## 1. Introduction

Fear is a primary emotion essential for safeguarding human integrity. When exposed to threatening or risky situations, the brain activates neural circuits that prepare the organism to respond adaptively. This response mechanism positions fear as a mental alarm that protects physical and psychological well-being [1,2]. Throughout the history of neuroscience, various efforts have been made to classify and characterize fear, primarily through neuroimaging techniques. However, electroencephalography (EEG) has opened new possibilities for both everyday applications and scientific research due to its high temporal and probabilistic resolution, combined with relatively low operational costs. These features make EEG an ideal tool for analyzing basic emotions such as fear [3].

The integration of EEG with artificial intelligence (AI) and machine learning (ML) techniques has gained particular relevance in recent years, enabling substantial progress in the automatic recognition of primary emotions. Despite these advances, most research has focused on emotions such as happiness, sadness, and anger, while fear detection via EEG signals remains an emerging area—primarily due to its potential applications in mental health, neuropsychology, cognitive monitoring, and intelligent alert systems [4,5].

Furthermore, there exists a wide variety of EEG devices, frequency bands, algorithms, and experimental paradigms for fear induction. This diversity underscores the need for a systematic review of the literature to identify the most effective AI/ML algorithms used in fear detection through EEG. It is also necessary to synthesize and analyze key findings and methodologies across studies.

To address the following research question: What are the most effective methods, algorithms, and configurations reported in the literature for detecting fear from EEG signals using artificial intelligence?. this systematic review synthesizes studies published over the last 25 years focusing on fear detection via EEG processed with AI and/or ML techniques. The PRISMA 2020 methodology was followed, using a structured search string that excluded studies relying on medical imaging or technologies other than EEG. This review critically examines emotion models, EEG devices, stimulation protocols, electrode placements according to the 10–20 system, and classification algorithms, emphasizing their performance and real-world applicability.

## 2. Methods, Techniques, and Instruments

### 2.1. Methodological Approach

This systematic review was conducted in accordance with the PRISMA 2020 guidelines (Preferred Reporting Items for Systematic Reviews and Meta-Analyses) (Supplementary Materials File S1), a widely accepted standardized framework designed to ensure transparency, replicability, and methodological quality in review studies [6]. PRISMA provides a clear structure for the identification, selection, evaluation, and synthesis of relevant studies, which helps minimize bias and ensure comprehensive coverage when addressing the research question.

### 2.2. Search Strategy

A structured search was conducted across three scientific databases: Scopus, Clarivate, and PubMed. The search string applied was as follows:

"fear detection" AND ("artificial intelligence" OR "machine learning") AND NOT (fnirs OR mri OR ct OR pet OR image)

This search string was designed to exclusively identify studies that applied artificial intelligence or machine learning techniques to the analysis of EEG signals for the purpose of emotion detection, excluding works based on neuroimaging modalities such as fMRI, tomography, or medical imaging.

### 2.3. Inclusion and Exclusion Criteria

This strategy excluded all studies that employed neuroimaging techniques, such as fNIRS, MRI, PET, etc., in order to ensure alignment with the study’s objective. Additionally, the following inclusion criteria were applied:

- Empirical studies and patents published between 2000 and 2025.
- Explicit use of EEG signals for fear detection.
- Application of AI and/or ML techniques.
- Abstract must clearly indicate that fear detection is performed using EEG and AI.
- Full-text availability.

The exclusion criteria were as follows:

- Studies focused on other basic emotions.
- Research that did not use AI/ML techniques.
- Works that relied exclusively on neuroimaging (fMRI, PET, etc.).
- Reviews or theoretical papers without experimental validation.

### 2.4. Selection Process

**Figure 1:**
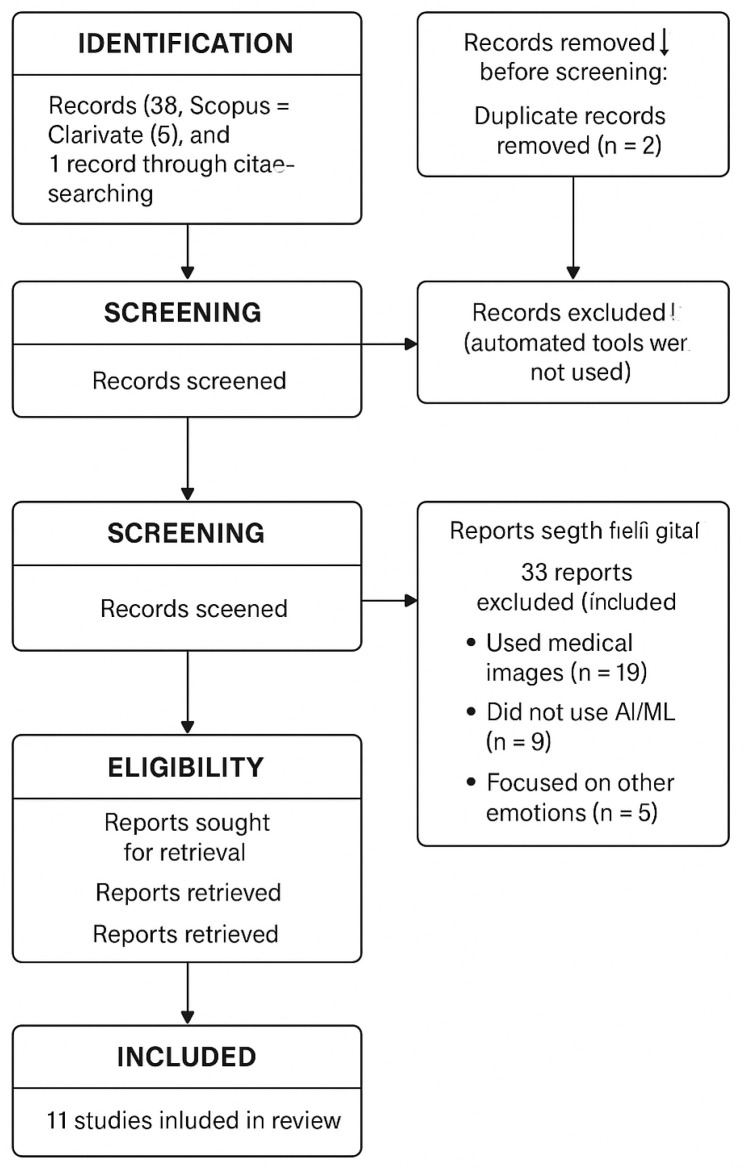
PRISMA 2020 flow diagram representing the study selection process for the systematic review on EEG-based fear detection using artificial intelligence. Generated with the assistance of ChatGPT 4o.

Initially, 45 studies were identified: 38 from Scopus, 5 from Clarivate, and 2 from PubMed. After removing duplicates (n=2) and excluding irrelevant records through manual screening (n=33), 43 full-text articles were retrieved. Of these, 33 were excluded for not meeting the thematic criteria. In the end, 11 studies were included for detailed analysis. This process is illustrated through a flow diagram following the PRISMA model (see Figure 1).

### 2.5. Data Extraction and Synthesis

The data extracted from each article included the following: algorithms used, model accuracy, hardware configuration, electrode placement, analyzed brainwave bands, stimulus duration, protocol type, database employed, and preprocessing techniques. The information was organized into comparative tables and analyzed using descriptive techniques and thematic categorization in order to identify patterns, trends, and gaps in the literature.

### 2.6. Quality Assessment and Risk of Bias

To ensure the methodological robustness and internal validity of the included studies, a qualitative assessment of risk of bias and overall study quality was conducted. Given the methodological heterogeneity among the reviewed studies—including experimental, observational, lab-based, simulated, and dataset-development designs—an ad hoc evaluation matrix was constructed based on adapted criteria from standard frameworks such as the AXIS protocol for observational studies and the PRISMA 2020 guidelines for systematic reviews.

The assessment considered ten key criteria:

- Clarity of the research objective and experimental design.
- Use of validated and replicable fear induction paradigms (e.g., videos, imagery, and immersive contexts).
- Quality of EEG signal acquisition (hardware type, the number of channels, and sampling rate).
- Appropriateness of signal preprocessing and feature extraction techniques.
- Use of AI/ML-based classifiers and their methodological transparency.
- Implementation of cross-validation or independent validation procedures.
- Sample representativeness and demographic description.
- Explicit ethical approval and participant consent procedures.
- Degree of transparency in reporting results and study limitations.
- Risk of bias due to small sample sizes, overfitting, or selective reporting.

Each study was independently assessed by at least two reviewers. Discrepancies were resolved through consensus. Based on the totality of these criteria, studies were categorized as having low, moderate, or high methodological risk.

Table 1 presents the full evaluation of the eleven included studies. The results indicate that most studies achieved moderate methodological quality, with consistent reporting of experimental design and ethical approval. However, several studies lacked independent validation procedures, comprehensive demographic reporting, or detailed justification of their fear induction protocols. Notably, studies relying on single-sensor EEG or introspective paradigms tended to score lower in signal robustness and ecological generalizability. Conversely, systematic reviews and multimodal database analyses demonstrated stronger methodological integrity and clearer articulation of bias mitigation strategies.

This analysis underscores the need for greater standardization in future research—particularly in participant sampling, reporting of model validation methods, and ethical transparency in affective computing applications.

## 3. Results

After applying the PRISMA methodological protocol and conducting a detailed analysis of the 11 selected studies, the relevant information was systematized into a series of comparative tables. These tables provide a structured overview of the main approaches, techniques, configurations, and findings reported in the literature related to fear detection using EEG signals and artificial intelligence.

Each table groups and summarizes the key aspects evaluated, including the algorithms used and their performance, the EEG devices employed, the available databases, the emotional models applied, the brain regions involved, the preprocessing methods, the EEG bands associated with fear, the duration of the experiments, and the stimulation protocols used. This organization facilitates comparison across studies and lays the groundwork for a critical discussion of current practices and their technological and neuroscientific implications.

In order to address the research question, 11 studies were analyzed following a rigorous systematic review process. The most relevant findings are presented and discussed in depth below based on the generated results tables.

### 3.1. Algorithms Used and Their Performance

Table 2 presents the most commonly used algorithms for classifying fear based on EEG signals. A clear preference for non-linear models is observed, particularly Support Vector Machines (SVMs), Decision Trees, Random Forest, and Convolutional Neural Networks (CNNs). These models have demonstrated their ability to handle complex relationships between physiological variables and emotional categories, outperforming traditional linear methods such as logistic regression.

The use of Decision Trees proved particularly effective in simulated environments. In the study by Turnip et al. (2024) [16]. the implementation of this algorithm within a flight simulator setting achieved an accuracy rate of 92.95%. This performance is partly explained by the model’s ability to make decisions based on logical thresholds and its ease in interpreting hierarchical patterns within non-linear datasets. The structural simplicity of this approach also makes it an attractive option for embedded systems.

On the other hand, Support Vector Machines (SVMs) stand out as one of the most robust and consistent algorithms in this field. Studies such as those by Krishna et al. (2019) [11] and Ishizuka et al. (2024) [15] showed that, when combined with proper feature extraction techniques (such as DWT or EMD) and signal denoising, this model can exceed 90% accuracy. Furthermore, the SVM offers a particular advantage in scenarios with medium-sized datasets as its margin-based function maximizes class separation with moderate computational cost. This makes it ideal for integration into portable systems and devices with limited computing capacity.

Deep learning algorithms, such as Convolutional Neural Networks (CNNs) and Deep Belief Networks (DBNs), have shown high potential, especially in studies involving large, well-labeled datasets. Vempati and Sharma (2023) [17] reported strong results using CNNs to detect emotions with high spatial dimensionality in EEG patterns. However, these approaches require large amounts of data to avoid overfitting and are more sensitive to the quality of emotional labeling. Additionally, their computational demands pose a barrier for deployment in continuous or real-time monitoring systems, particularly in populations outside the laboratory setting.

In summary, non-linear algorithms such as the SVM and Random Forest offer an optimal combination of performance, scalability, and technical feasibility for integration into practical solutions. Their flexibility to adapt to different EEG acquisition schemes, along with their relatively low computational cost, makes them preferred candidates for implementation in clinical settings, households, or mobile environments. Moreover, their interpretability and algorithmic transparency add additional value in contexts where explainability is essential, such as mental health applications or elderly monitoring.

### 3.2. EEG Hardware Configurations

Table 3 shows a marked diversity in the EEG devices used across the analyzed studies, reflecting a balance between the need for neurophysiological precision and applicability in real-world settings. On one hand, clinical-grade equipment such as the Biosemi ActiveTwo and Neuroscan SynAmps RT stand out for their high channel density (64 to 128 electrodes), superior sampling frequency (up to 2048 Hz), and high-fidelity signal acquisition. These features make them the gold standard for controlled neuroscience research, where precise cortical localization and complex topographical analysis are required [7,9].

However, their use also implies significant limitations: high cost, low portability, lengthy calibration procedures, and the need for a specialized technical environment. These conditions restrict their practical viability in out-of-laboratory applications, such as in homes or community hospitals.

On the other hand, portable and commercial-grade devices such as the Emotiv Epoc+, MyndPlay, and Emotiv Insight, while offering lower resolution (5 to 14 electrodes and sampling frequencies of 128–256 Hz), excel in ergonomic design, ease of use, low cost, and wireless connectivity. Studies like that of [14] demonstrated that, with the use of robust signal processing pipelines—including bandpass filtering, artifact removal via ICA, and feature extraction through DWT or EMD—it is possible to achieve classification rates above 85%, even under uncontrolled environmental conditions.

Additionally, technologies like OpenBCI offer an intermediate option: open-source, modular devices with expandability up to 16 channels, allowing for custom configurations based on experimental paradigms. The authors of [15] leveraged this flexibility in mixed environments, combining visual stimulation with multiband analysis of brain signals and yielding promising results in both accuracy and adaptability.

The choice of EEG hardware should not be seen as a purely technical decision but rather as a strategic one that considers the type of user, the context of use, and the system’s computational requirements. Portability, autonomy, and ease of placement become key factors. This implies that systems based on consumer-grade hardware must compensate for their lower resolution through a more rigorous methodological design in terms of preprocessing, electrode placement, and personalized calibration.

Despite their viability, a lack of comparative studies across different types of hardware applied to the same protocol was identified. This gap limits the ability to establish objective guidelines for selecting devices based on performance, cost, and usability. Future research should address this comparison using standardized methodologies that evaluate variables such as accuracy, latency, signal stability, artifact robustness, and user acceptance.

In summary, the review suggests that portable EEG devices can be not only adequate but even preferable for emotion detection applications outside the laboratory, provided that their technical integration is supported by a systemic approach that encompasses the entire acquisition, processing, and classification pipeline. In this way, the implementation of accessible, efficient, and scalable solutions in social, clinical, and educational contexts is greatly enhanced [17,18].

### 3.3. Databases and Emotional Stimulation Paradigms

Table 4 and Table 5 show that most of the studies included in this review relied on public databases such as DEAP, DREAMER, and GAMEEMO, which are designed for emotion classification through audiovisual stimuli. These datasets typically use video clips or images labeled with valence and arousal scales (e.g., SAM–Self-Assessment Manikin), facilitating reproducibility and comparison across algorithms and models [14,19]. However, they present methodological limitations when focusing on a specific emotion like fear as they are often centered on general emotional states or lack a sufficient number of fear-specific samples.

In contrast, studies that developed personalized databases [7,16] achieved greater experimental control over stimulus quality and intensity. Turnip et al. used a highly realistic flight simulator as an emotional induction environment, while Proverbio and Cesati designed a protocol based on guided autobiographical recall. These approaches enabled the capture of more intense, distinguishable, and emotionally congruent EEG responses, resulting in more accurate models with lower error rates. Personalized datasets also allowed the adjustment of contextual variables such as stimulus duration, breathing rhythm, recovery time, and environmental control—factors rarely considered in public datasets.

Regarding stimulation paradigms, it was found that dynamic audiovisual stimuli, especially high-emotional-load videos or interactive simulations, were the most effective at inducing sustained fear. The use of emotional video games, flight simulators, or immersive narratives triggered prolonged activation of brain networks associated with fear, facilitating the identification of specific patterns in the beta and gamma bands [14,16]. These configurations more accurately replicate real-world conditions of threat or uncertainty, offering a more ecologically valid environment than passive exposure to images or sounds.

In contrast, studies using static images or acted phrases, such as those in the CREMA-D dataset [12], showed lower effectiveness in inducing specific fear. This is because fear often requires contextualization and temporal buildup to fully develop at the neurophysiological level. Moreover, brief stimuli may elicit attentional or reflexive responses but do not necessarily activate the limbic circuits involved in fear processing.

The results suggest that the choice of data source and emotional stimulation paradigm has a direct impact on the quality of recorded EEG signals and, consequently, on the performance of classification algorithms. While public datasets are useful for model validation and facilitating comparisons between studies, their ability to induce authentic fear is limited due to the low affective load of stimuli and their generalist focus.

In contrast, personalized datasets allow for greater emotional specificity, experimental control, and adaptability to user context. This is especially relevant for applications in sensitive populations, where induction must be ethical, gradual, and safe. Additionally, naturalistic and immersive paradigms yield more sustained responses, reducing the need for extremely short observation windows or excessive processing to detect subtle changes.

One identified limitation is the lack of standardization in emotional stimulation protocols, which prevents direct comparisons between studies. Future work should consider building open repositories of fear-validated emotional stimuli, as well as developing tools to calibrate stimulus intensity based on the emotional profile of the participant.

### 3.4. Brain Regions and Electrode Placement

Table 6 shows that electrodes placed over the frontal (Fp1, F3, Fz) and temporal (T4, T7, T8) regions of the scalp exhibit the highest sensitivity to brain activation patterns associated with fear. These findings are consistent with the neuroscientific literature, which identifies the dorsolateral prefrontal cortex, orbitofrontal cortex, and medial temporal lobe structures—particularly the amygdala—as key nodes in the brain’s fear network [4,5]. Activation of these areas is related both to the cognitive appraisal of threatening stimuli and to the physiological preparation for action (fight, flight, or freeze).

The reviewed studies reinforce this functional association. For example, Proverbio et al. (2024) [7] observed that activity recorded in F3 and Fp1 significantly increased during guided emotional recall tasks focused on fear-related memories, while Ishizuka et al. (2024) [15] found strong correlations between increased gamma-band power in T7 and subjective fear responses induced by simulations. Likewise, Subasi et al. (2021) [18] documented sustained increases in frontal electrode activity in response to aversive stimuli, using wavelet transform and multiband segmentation.

From a practical and technological perspective, these findings have key implications. Focusing on the frontotemporal regions allows for the design of EEG systems with a reduced number of channels without compromising the quality of emotional analysis. This optimization not only lowers equipment costs but also simplifies system design, improves device ergonomics, and shortens calibration times—crucial factors for implementing portable or home-based solutions.

Furthermore, frontal regions are more anatomically accessible, facilitating sensor placement without the need for specialized technical assistance. In contexts such as monitoring elderly individuals or patients with reduced mobility, this represents a significant advantage. This choice may also enhance user acceptance by avoiding invasive or uncomfortable configurations.

However, some limitations were also identified. Certain studies do not explicitly report the electrode locations with the highest activation or fail to conduct detailed topographic analyses. This lack of standardization in result presentation limits the ability to generalize precise recommendations for optimal fear-related electrode placement.

Finally, the consistent activation of frontotemporal areas may be mediated by emotional lateralization, a property still under debate in the neurophysiological literature. Some studies suggest that fear is more strongly expressed in the right hemisphere, particularly in the right frontotemporal region [20], which could have implications for designing asymmetric or adaptive emotional monitoring systems.

### 3.5. Dominant Brainwave Frequency Bands

Table 7 consolidates the most relevant findings on the activity of different brain frequency bands during the emotional experience of fear. Consistently, the reviewed studies report an increase in beta (13–30 Hz) and gamma (30–100 Hz) band activity, along with a decrease in alpha band (8–12 Hz) activity, in response to stimuli perceived as threatening. These results align with the neuroscientific literature, which links the beta band to cognitive alertness, active processing, and motor preparation [21], while the gamma band is associated with emotional perception integration, selective attention, and the encoding of intense memories [22].

The reduction in alpha power, particularly in frontal and occipital regions, indicates a shift from resting or relaxed states to a vigilance or threat anticipation response. Klimesch [23] has emphasized that alpha activity is strongly related to cortical inhibition processes; its suppression suggests disinhibited access to relevant sensory information, which is adaptive in threatening contexts. In studies like [15], pronounced alpha desynchronization was observed during fear recognition tasks, alongside gamma band increases, especially in temporal electrodes.

Regarding the theta band (4–7 Hz), although less frequently reported, some studies mention its activation during deep emotional processing tasks, particularly in the medial cortex and hippocampus. This may relate to contextual memory of fear or the reactivation of past experiences [24].

From a functional perspective, beta and gamma bands emerge as key candidate biomarkers for fear detection through EEG, given their sensitivity to intense emotional activation and their correlation with attentional and perceptual processes linked to danger. However, effective utilization requires the application of adaptive multiband feature extraction techniques, such as Discrete Wavelet Transform (DWT) or Empirical Mode Decomposition (EMD), which enable the isolation and quantification of spectral energy with greater temporal resolution.

Moreover, gamma signals—due to their high frequency and low voltage—are especially vulnerable to artifacts and muscular noise. For this reason, studies with better results applied specialized filtering, adaptive window segmentation, and the combination of spectral and spatial analysis (e.g., topographic mapping) to ensure reliability [7,18].

In practical environments, such as emotional monitoring in older adults or the implementation of real-time alert systems, this information is highly valuable. The use of these bands as real-time indicators could enable the deployment of low-latency emotional inference models, particularly when combined with sustained emotional stimulation protocols. Additionally, alpha suppression could serve as an initial activation signal in systems requiring a baseline threshold before triggering an alert.

Nevertheless, a gap is noted in the literature regarding inter-individual variability of emotional bands, as well as their dynamic interaction. Future research should explore multivariate and longitudinal models analyzing the simultaneous coactivation of bands in response to different types of fear (acute, anticipatory, and contextual) and across various populations (young vs. older adults; clinical vs. healthy).

### 3.6. Data Preprocessing

Table 8 highlights that EEG data preprocessing is a critical stage in the analysis pipeline, with a direct impact on machine learning quality and emotional classification accuracy. The reviewed studies applied a diverse combination of techniques, including: band-pass filtering (typically between 0.5 and 50 Hz), artifact removal for ocular or muscular movement (using manual or automated methods such as ICA), segmentation into temporal windows, and advanced transformations such as Empirical Mode Decomposition (EMD), Discrete Wavelet Transform (DWT), or Fourier Transform.

For instance, ref. [18] demonstrated that applying DWT followed by dimensionality reduction through PCA significantly improved SVM classifier accuracy while reducing redundant noise. The authors of [15] used EMD to extract intrinsic signal components and combined these vectors with logistic regression and supervised learning algorithms, achieving up to 12% improvements in accuracy metrics compared to unprocessed signals. Additionally, studies like [14] implemented fully automated cleaning and normalization pipelines, enabling the use of portable EEG in uncontrolled conditions, such as emotional gaming at home.

The data suggest that the effectiveness of a fear detection system does not rely solely on the classification algorithm but rather on the quality of the extracted features, which in turn depend on robust preprocessing. This implies that any real-life-oriented system should include a standardized and automated EEG signal processing pipeline. Ideally, this pipeline should address the following: (i) physiological artifact removal, (ii) adaptive contextual segmentation, (iii) multiband feature extraction (wavelet and EMD), and (iv) dimensionality reduction to enhance computational efficiency. Ignoring this stage compromises system reliability and may result in false positives or negatives, especially in noisy environments or with complex brain signals.

### 3.7. Emotion Model in Fear Detection

Table 9 presents the analysis of emotion models used across studies reveals a methodological diversity that significantly impacts the quality of fear classification. The basic emotions model by Ekman [7,8,15] has proven effective for direct classification tasks due to its simplicity and its alignment with well-defined neurophysiological patterns. However, its discrete approach may limit the detection of emotional nuances.

In contrast, the circumplex model by Russell, often used with scales like SAM [9,14,17], allows for a more nuanced representation of affective states across valence and arousal dimensions. This model offers higher sensitivity to subtle changes but requires more complex computational approaches.

The five-level fear model [16] stands out as a middle ground, enabling finer-grained classifications in controlled environments such as flight simulators. Meanwhile, guided acting approaches provide experimental control but reduce emotional authenticity, whereas naturalistic induction [13] improves ecological validity at the cost of greater variability and noise.

In summary, there is no universally superior model. The choice must align with system objectives, experimental context, and target population. Discrete models are suitable for simplified clinical settings, while dimensional or graduated models offer greater sensitivity for adaptive or long-term monitoring systems.

### 3.8. Stimulus Duration and Experimental Context

Table 10 shows that the duration of the emotional stimulus and the context in which it is presented are key factors in the effectiveness of fear detection using EEG. Studies with prolonged protocols, such as [16], which used flight simulators in sessions lasting up to 30 min, achieved greater stability in EEG patterns associated with fear. Similarly, alakus et al. (2020) [14] employed 5-min emotional video game sessions, generating continuous and contextualized interaction with the stimulus, which enabled the detection of more robust brain responses.

In contrast, studies such as Balan et al. (2019) and Subasi et al. (2021) [9,18], which worked with databases like DEAP (using videos approximately 1 min long), showed acceptable results but with higher variability across subjects. Even shorter studies, such as those using acted clips from the CREMA-D dataset (1–3 s), presented low emotional resolution due to the brain’s limited ability to generate an intense affective response within such brief time windows.

Stimulus duration directly influences the EEG system’s ability to capture emotional dynamics. The longer the stimulus, the greater the resulting brain activity—often with higher amplitude. For this reason, it is said that prolonged stimuli allow the observation of the full cycle of emotional processing: anticipation, perception, cognitive evaluation, and physiological response. Additionally, extended protocols reduce the likelihood of artifacts caused by sudden noise or startle reactions.

In practical applications, it is recommended that systems include sustained analysis windows or prolonged/interactive stimuli to ensure that brain responses are consistent enough to feed a classification algorithm with high reliability. Consequently, immersive environments, extended narratives, or real-time interaction not only increase system sensitivity but also enable the segmentation of more stable and less noise-prone signals. This is essential for real-time applications where decisions must be based on continuous and cumulative evidence.

### 3.9. Age Influence on EEG Patterns Associated with Fear

The age composition of the samples used in the studies included in this review presents significant methodological and conceptual implications for the detection of fear using EEG signals and artificial intelligence. As shown in Table 11, a substantial proportion of the studies focused exclusively on young adults, primarily university students, with little to no representation of other stages of the lifespan. This homogeneous sampling pattern was observed in at least seven of the eleven reviewed studies, while only two investigations, specifically those by Cao et al. and Sneddon et al., reported demographically diverse samples. Additionally, in two cases, the participants’ ages were not explicitly reported, representing a critical methodological omission in neurophysiological studies, where age constitutes a key modulator of brain activity.

Age is a decisive factor in the functional configuration of the brain, particularly in frontal regions, which are central to emotional processing. In this context, multiple studies have documented that the electrical dynamics of the brain, as measured by electroencephalography, change substantially across the lifespan. Cortical maturation, synaptic reorganization, and variations in functional connectivity directly influence the morphology of EEG signals and their association with affective and cognitive states [25,26,27].

A central metric in emotional EEG evaluation is frontal alpha asymmetry, particularly in electrodes F3 and F4, which has been consistently associated with negatively valenced emotions such as fear. The presence of right-hemisphere alpha suppression in response to threatening stimuli has been interpreted as a marker of cortical activation linked to withdrawal motivation and aversive processing [28,29]. This suppression—commonly referred to as “alpha suppression”—acts as a neurophysiological indicator of emotional engagement. However, the strength and consistency of this response are not uniform across ages. Studies such as Marshall et al. [25] have shown that during early childhood and adolescence, frontal asymmetry patterns are not yet fully developed, limiting their reliability as emotional markers at those stages. Conversely, research by Christou et al. [27] suggests that in older adults, interindividual variability in EEG responses to emotional stimuli increases, potentially reflecting neurobiological aging and cognitive compensation mechanisms.

This body of evidence highlights the need to treat age not merely as a descriptive variable but as a critical parameter that must be explicitly incorporated into algorithmic models for emotion detection. Using artificial intelligence models trained exclusively on data from young adults entails the risk of generating predictive biases when applied to different populations. This concern is far from trivial as the field of affective neurotechnology is advancing toward clinical, educational, and wellness applications, where model validity depends directly on its capacity to represent human brain diversity.

It is worth noting that this concern has already been addressed in other areas where EEG and AI are applied. For example, in automated diagnosis of depressive disorders, variables such as age and gender have been shown to affect brain connectivity patterns and nonlinear EEG features, thereby influencing classifier performance [30]. Similarly, in studies of mild cognitive impairment, the activity of alpha rhythm sources varies as a function of age and correlates with cerebral metabolic state [31]. Even in brain–computer interfaces designed for navigation or rehabilitation, user-specific calibration has proven essential to improve model accuracy [32].

In light of these findings, it becomes evident that the limited age diversity in studies on fear detection using EEG constitutes one of the major methodological weaknesses in the field. Including participants of different age groups would not only enhance the generalizability of models but also allow for a more rigorous exploration of neurophysiological emotion variations across the lifespan. Furthermore, integrating such data into the training and validation of AI systems could open the door to developing more robust, personalized, and ethically aligned classifiers that better reflect the complexity of the human emotional brain.

### 3.10. Hybrid and Multimodal Approaches to EEG-Based Fear Detection

The integration of electroencephalography (EEG) with artificial intelligence has catalyzed the emergence of hybrid systems for fear detection. As shown in Table 12, the reviewed studies collectively illustrate a transition from conventional, retrospective approaches to adaptive, multimodal systems capable (at least in part) of real-time emotion tracking. These systems vary in technical sophistication, methodological design, and translational maturity but together delineate the contours of an evolving research frontier in affective neuroscience and computational modeling.

Broadly speaking, the studies can be categorized into two main trajectories. The first relies on pre-established, emotion-labeled EEG datasets such as DEAP (e.g., Bălan et al. (2019) and Krishna et al. (2019), [9,11]), which provide high experimental control but limit ecological validity and emotional authenticity. These works employ multichannel EEG combined with peripheral physiological signals (e.g., GSR, EMG, temperature, and respiration), extracting complex features such as fractal dimensions, entropy, and Hjorth parameters. Classifiers such as Extreme Learning Machines (ELMs) and Support Vector Machines (SVMs) yielded classification accuracies exceeding 85%, particularly when leveraging multimodal input. However, the reliance on offline labeling post-stimulation introduces constraints regarding temporal precision and undermines their real-world adaptability.

Conversely, studies by Ishizuka et al. (2024) [15] and de Man and Stassen (2017) [8] pursued experimental designs tailored to the participant, focusing on emotionally relevant stimuli and personalized emotion tagging. Ishizuka et al. employed an 8-channel EEG system, combined with empirical mode decomposition (EMD) and a post-stimulation interview for labeling emotional segments. Despite the small sample size (*n* = 5), their system achieved a subject-dependent classification accuracy of 87.7%, demonstrating feasibility for lightweight, portable applications. de Man et al. [8], on the other hand, used a single-channel EEG with real-time emotional self-reporting via a rotary potentiometer, achieving meaningful differentiation between calm and fear states through high-beta and low-gamma band analysis. These studies, although methodologically less complex, offer valuable insight into the construction of low-cost systems with potential for continuous, subjective emotion assessment.

Kim et al. (2019) [10] represent a middle-ground approach, exploring perceptual fear in urban settings through mobile EEG and static nightscape images. Their findings—an increase in beta activity and decrease in alpha rhythms in response to isolated, human-free urban scenes—underscore the influence of environmental context in modulating fear responses. Although not based on deep learning nor real-time classification, this work broadens the scope of fear induction paradigms and supports applications in environmental psychology and urban design.

From a translational standpoint, the study by Turnip et al. (2024) [16] is particularly noteworthy. Utilizing a clinical-grade EEG system (Mitsar 202) with 19 channels, they simulated critical landing phases in a flight simulator and achieved 92.95% accuracy in classifying five levels of fear using Decision Tree classifiers. The system integrates signal features from alpha, beta1, beta2, and gamma bands, highlighting the feasibility of EEG-based fear recognition in complex, cognitively demanding tasks. Nevertheless, the lack of physiological signal fusion, real-world deployment, and inter-subject validation indicates that the system remains in a pre-implementation phase.

Algorithmically, the reviewed studies confirm the superiority of hybrid and deep learning models (such as CNN-LSTM, TQWT + ELM, and EMD + SVM) over traditional classifiers. These architectures benefit from their capacity to integrate spatial and temporal features, capturing nonlinear dynamics of affective responses. However, the absence of cross-dataset validation and the frequent use of single, pre-labeled databases limit the generalizability of these results. Moreover, the field continues to lack standardized evaluation metrics and shared benchmarks, hindering comparative analysis and reproducibility.

Equally concerning is the persistent absence of ethical safeguards. None of the reviewed studies addressed critical issues such as neural data protection, dynamic consent models, or algorithmic fairness—despite operating on neural correlates of one of the most evolutionarily significant and clinically sensitive human emotions. Given that fear responses are deeply modulated by trauma, context, and sociocultural background, the lack of ethical oversight is not only a methodological shortcoming but also a structural gap in affective computing research. Future systems must prioritize equitable design, user-centered calibration, and participatory frameworks to avoid replicating or amplifying bias under the guise of technological neutrality.

Overall, the reviewed systems reflect a field in active maturation, combining algorithmic innovation with increasingly ecologically valid paradigms. Yet, key gaps remain—methodologically (e.g., longitudinal validation and inter-subject replicability), operationally (e.g., testing in real-world environments), and socio-technically (e.g., ethical deployment and user diversity). Bridging these gaps requires coordinated interdisciplinary efforts across neuroscience, engineering, human–computer interaction, and neuroethics.

Only through such integration can EEG-based fear detection systems evolve from high-performing prototypes into socially responsible technologies, capable of serving health, safety, and human well-being without compromising personal dignity or cognitive autonomy.

### 3.11. Applicability of Hybrid EEG Systems in the Detection of Other Emotions

While this review has focused on fear detection through EEG signals, it is important to note that these technologies have also demonstrated significant potential in identifying other complex emotional states, such as depression. Several studies have employed hybrid systems that combine EEG with artificial intelligence algorithms and multimodal approaches, yielding promising results in both diagnostic accuracy and clinical characterization.

For instance, Yasin et al. [33] conducted a comprehensive review of methods integrating audiovisual and EEG signals with machine learning algorithms such as SVM, KNN, and CNN. Their analysis concluded that multimodal systems significantly enhance depression detection and relapse prediction. However, they highlighted limitations such as subject variability and the need for methodological standardization.

In a subsequent study, the same group evaluated the effectiveness of active and passive EEG paradigms, combined with classifiers (SVM, Decision Trees, and logistic regression) and clustering techniques (t-SNE and kernel PCA). Their hybrid system achieved 100% accuracy using Decision Trees in distinguishing depressed individuals from controls, emphasizing the relevance of active paradigms in detecting cognitive deficits associated with depression [34].

Similarly, Yasin et al. [35] proposed a hybrid model based on a CNN-LSTM architecture applied to quadriplegic patients, achieving 98% accuracy in depression relapse detection. This study is pioneering in integrating EEG and clinical data for this specific population, demonstrating the versatility of these techniques in challenging clinical contexts.

Finally, their systematic review focused on EEG-based diagnosis of major depressive disorder and bipolar disorder using artificial neural networks (ANN, CNN, RNN, and DBN). The review highlights the effectiveness of hybrid models leveraging nonlinear features and biomarkers while pointing out the need for dataset standardization and better handling of EEG signal noise [36].

These findings reinforce the notion that EEG-based systems, especially when combined with hybrid AI models, are not only useful for detecting fear but also offer a robust framework for identifying emotional disorders such as depression, opening new avenues for clinical and technological research.

### 3.12. Synthesis and Response to the Research Question

Based on the comprehensive analysis of the 11 studies included in this systematic review, key elements were identified that define an optimal configuration for fear detection from EEG signals using artificial intelligence. Together, these findings provide a well-supported answer to the research question.

First, it is confirmed that non-linear algorithms such as Support Vector Machines (SVMs) and Decision Trees offer a superior balance between accuracy, efficiency, and ease of implementation. Their performance significantly improves when trained on preprocessed data labeled using consistent emotional models (e.g., Ekman or Russell), achieving accuracies above 90% under controlled conditions. Moreover, these models have demonstrated greater robustness to inter-individual variability—a critical aspect for diverse populations such as older adults.

Second, the importance of the frontotemporal brain regions in fear encoding is emphasized, which allows for a reduced electrode configuration without sacrificing diagnostic quality. This observation is fundamental for the design of portable systems that do not rely on complex clinical setups. The use of commercial EEG devices such as Emotiv or MyndPlay is justified in this context, provided they are used with specific placement protocols and standardized signal-cleaning methods.

Additionally, the stimuli used to induce fear are a key component of the system architecture. Prolonged simulations, emotional video games, and immersive scenarios generate more distinct and sustained neural patterns compared to short clips or artificial stimuli. This leads to higher classifier reliability, fewer false positives, and greater sensitivity to gradual emotional state changes.

Furthermore, multiband feature extraction focused on gamma and beta activity, along with alpha suppression, proves to be a robust practice for capturing brain signals related to fear. When combined with techniques like DWT or EMD, this enables the construction of enhanced graphical representations of EEG signals, facilitating detection and classification by learning algorithms.

Ultimately, the cross-cutting element that determines system effectiveness is the integration of all these techniques into a coherent, automated, and experimentally validated pipeline. It is not enough to select powerful algorithms or appropriate sensors—the synergistic articulation between stimulus, signal acquisition, preprocessing, feature selection, and inference defines the real capacity of a system to detect fear with accuracy, stability, and in real time.

In summary, the reviewed studies converge on a comprehensive methodological model where the combination of accessible hardware, realistic emotional stimulation protocols, advanced neuroinformatics processing, and non-linear classification models offers a more effective and applicable approach for automatic fear detection in clinical, preventive, and assistive contexts.

### 3.13. Ethical Considerations

One of the most relevant findings of this review is the very limited number of studies that explicitly address fear detection using electroencephalography (EEG) and artificial intelligence. Only eleven studies met the inclusion criteria, which not only reflects the emerging nature of this research area but also underscores the presence of multiple ethical, methodological, and technological barriers that hinder its development. These include the emotional risks involved in inducing fear, the complexity of obtaining informed consent in neuroemotional contexts, and the widespread absence of robust ethical protocols for the collection, interpretation, and use of brain data linked to affective states.

Inducing fear experimentally entails significant emotional and psychological risks. Unlike positive or neutral emotions, fear can activate traumatic memories, trigger acute anxiety episodes, and cause prolonged psychological discomfort, especially among individuals with clinical backgrounds or emotional vulnerability [4,5]. This affective load explains why many ethics committees restrict or reject protocols involving the direct induction of fear, thus limiting the number of viable studies. However, this also reveals an unresolved tension: it is necessary to study fear as a relevant clinical marker but under conditions that guarantee the emotional safety and integrity of participants.

Among the reviewed studies, a general absence of differential ethical frameworks was identified. Few works reported specific strategies to ensure emotionally contextualized informed consent. None offered emotional crisis management protocols or post-exposure psychological support. Similarly, there is a lack of procedures for handling sensitive data, such as EEG patterns associated with fear responses, which could be used—especially in unregulated settings—for emotional profiling or inference without the subject’s awareness or consent.

This gap becomes even more critical when considering the demographic composition of the samples. As shown in Table 11, most studies relied on homogeneous populations: young, university-educated adults, with minimal or no ethnic, age, or clinical diversity. The absence of demographic representation not only restricts generalizability but also increases the risk of algorithmic bias, exclusion, and classification errors when these models are applied to broader populations. From an ethical standpoint, this constitutes a problem of epistemic and distributive justice: technologies are being built based on data from a narrow segment of humanity, while their deployment is intended to be global and inclusive.

The risk is not merely technical. If algorithms are trained to detect emotional patterns based on non-diverse EEG data, they are likely to produce false positives or negatives, particularly in individuals whose neural responses vary due to genetic, developmental, or cultural factors. In clinical contexts, this could lead to misdiagnoses or stigma. In occupational or security environments, it could result in discriminatory practices or unauthorized emotional surveillance. The absence of safeguards exposes participants to unregulated and ethically ambiguous uses of their brain activity—a form of technological vulnerability that current legislation has yet to address.

Likewise, the type of stimulus used to induce fear is ethically and methodologically critical. As suggested in the literature [37], the use of controlled, progressive, and symbolic environments, such as virtual reality or abstract representations, is necessary to elicit fear without causing trauma. Poorly designed or overly intense stimuli cannot only lead to emotional harm but also compromise the ecological validity of the EEG signal. An artificially induced emotional state using unrealistic or irrelevant stimuli will generate atypical physiological responses, thus distorting the emotional brain signature and affecting the accuracy of AI models.

While some studies have explored fear detection in vulnerable populations—such as individuals with psychiatric conditions or quadriplegia—few have implemented differentiated ethical protocols. These populations require reinforced levels of consent, support, and protection, given their potential cognitive or communicative limitations. This is especially relevant when employing artificial intelligence, a technology often opaque to the general public and difficult to interpret without technical expertise.

Taken together, this review reveals a structural ethical gap in the development of EEG- and AI-based fear detection systems. Addressing this gap requires going beyond mere technical validation or institutional approval. It demands the integration of ethical principles from AI research (e.g., explainability, algorithmic fairness, and non-maleficence) as well as neuroethical frameworks sensitive to human diversity. Only in this way can we develop technologies that are not only effective but also fair, transparent, safe, and socially legitimate.

## 4. Limitations and Future Research Directions

Despite the significant advances identified in this review, multiple gaps remain that must be addressed to move toward more robust, generalizable, and ethically responsible EEG-based fear detection systems. Based on the systematic analysis conducted, including methodological quality assessment and in-depth review of hybrid models, the following limitations and future directions are proposed:

- Scarcity of fear-specific and high-quality datasets: Most available EEG databases (e.g., DEAP, DREAMER, and MAHNOB-HCI) were not designed to isolate fear as a discrete emotional category, nor do they contain ecologically valid or culturally sensitive fear stimuli. The limited availability of rigorously labeled, demographically diverse datasets significantly hampers the training of fear-specific models. Future research should focus on developing datasets that are not only fear-centered but also representative in terms of age, ethnicity, and clinical background and that adhere to rigorous ethical and technical standards.
- Lack of ecological validity and real-world validation: A significant proportion of studies were conducted in highly controlled laboratory environments. The absence of real-life scenarios and contextual variability weakens the applicability of the models. Future research should prioritize in situ experimentation in real environments such as clinics, homes, or public spaces, particularly involving older adults or neurodiverse individuals, to assess model robustness under natural movement, noise, and affective variability.
- Non-standardized emotional induction protocols: There is wide heterogeneity in the type, intensity, and delivery of fear stimuli (e.g., virtual reality, videos, and mental recall), which limits cross-study comparability. Moreover, some stimuli may trigger artificial or insufficiently authentic fear responses. Standardization is urgently needed in stimulus design, incorporating validated, progressive, and emotionally safe paradigms with clear ethical oversight.
- Insufficient exploration of hybrid and multimodal systems: Although the recent literature confirms the value of combining EEG with other physiological or behavioral modalities (e.g., facial EMG, heart rate, and thermal imaging), most studies rely on EEG alone. Future work should investigate fusion strategies (early, late, and deep-learning-based) to improve accuracy, reduce signal artifacts, and increase robustness in naturalistic contexts.
- Lack of personalization and neurophysiological adaptability: EEG responses to fear vary significantly depending on age, gender, psychological profile, and brain maturity. However, few models incorporate mechanisms for dynamic adaptation or personalized calibration. Systems should be capable of adjusting parameters based on individual asymmetry patterns (e.g., alpha suppression and frontal asymmetry), incorporating incremental learning and adaptive thresholds to better reflect intersubject variability.
- Absence of longitudinal or low-latency real-time applications: The current state of research lacks continuous monitoring systems capable of detecting gradual changes in fear-related neural markers. Future models should be designed to support longitudinal tracking (e.g., for preventive mental health), with real-time performance, low latency, and high temporal sensitivity to allow timely and actionable responses.
- Limited diversity in participant samples and risk of algorithmic bias: The reviewed literature reveals a strong bias toward young, neurotypical, university-educated populations, with minimal inclusion of older adults, children, or individuals with affective or cognitive disorders. This homogeneity limits model generalizability and increases the risk of systematic errors when applied to marginalized or vulnerable populations. Future studies must emphasize inclusive sampling and stratified validation protocols.
- Ethical oversight and contextual complexity: Many studies fail to address core ethical considerations such as data privacy, emotional safety, explainability, and informed consent in emotionally vulnerable settings. In addition, the absence of ethical strategies for post-experiment support or emotional debriefing poses potential harm to participants. Future research should adopt interdisciplinary frameworks that integrate AI ethics, neuroethics, and distributive justice to ensure that technological progress is accompanied by ethical rigor and societal responsibility.
- Low scientific output does not imply low importance: The limited number of articles found in this review reflects the ethical, methodological, and regulatory challenges of working with fear as a target emotion, not its irrelevance. On the contrary, fear is a critical early indicator of affective disorders, cognitive decline, and psychological trauma [38]. Thus, the scarcity of studies reinforces the urgency of systematic, ethically approved investigations that can establish a solid foundation for future clinical and social applications.

## Figures and Tables

**Table 1:** Methodological evaluation and risk of bias of the included studies.

Authors and Year	Clear Objective	Appropriate Design	Representative Sample	EEG Signal Quality	Fear Paradigm Validated	AI Reported	Cross-Validation	Ethical Approval	Bias Risk
Proverbio et al. (2024) [7]	Yes	Yes	Partial (*n* = 20, undergraduate students)	High (128 channels, artifact removal)	Yes (pictograms with prior evidence)	Not explicit (BCI mentioned)	No	Yes (ethics approval and informed consent)	Moderate
de Man & Stassen (2017) [8]	Yes	Yes (videos and subjective self-assessment)	Partial (*n* = 30, students and university staff)	Low–medium (single-channel BrainLink EEG)	Yes (validated videos)	No	No	Yes (anonymous data and informed consent)	Moderate
Bălan et al. (2019) [9]	Yes	Yes (DEAP dataset focused on fear)	Yes (32 participants from DEAP)	High (32-channel preprocessed EEG)	Partial (DEAP emotion dimensions)	Yes (naive Bayes, SVM, KNN, LDA, MLP)	Yes (10-fold)	Yes (public dataset, preapproved)	Low
Mintai Kim et al. (2019) [10]	Yes	Yes (visual image exposure paradigm)	Partial (*n* = 30, limited demographic details)	Medium (8-channel EEG with preprocessing)	Partial (fear inferred via perception)	No (only ERP/statistical analysis)	No	Yes (institutional ethics approval)	Moderate
Krishna et al. (2019) [11]	Yes	Yes (emotion classification using DEAP)	Yes (DEAP dataset with 32 participants)	High (32 channels, well-preprocessed)	Partial (dimensional tagging, not explicit fear)	Yes (MLP, Random Forest, SVM)	Yes (10-fold cross-validation)	Yes (ethical use of DEAP dataset)	Low
Cao et al. (2014) [12]	Yes	Yes (multimodal emotion dataset creation)	Yes (91 actors of various ages and backgrounds)	Not applicable (no EEG, only AV and facial data)	Yes (acted fear, externally validated)	No (only dataset design)	No (not applicable)	Yes (informed consent of actors)	Moderate
Sneddon et al. (2012) [13]	Yes	Yes (multimodal data with interviews)	Yes (20 culturally diverse participants)	Not applicable (audio/video facial data)	Yes (fear elicited via validated stimuli)	No (no modeling, only data creation)	No (not applicable)	Yes (detailed informed consent process)	Moderate
Alakus et al. (2020) [14]	Yes	Yes (EEG game-induced emotion dataset)	Partial (28 young participants, low diversity)	Medium (Emotiv EPOC, 14 channels)	Partial (labels inferred via gameplay)	No (dataset creation only)	No (no classification analysis)	Yes (ethics approved)	Moderate
Ishizuka et al. (2024) [15]	Yes	Yes (fear elicitation and SVM classifier)	Limited (*n* = 6, only 5 usable EEG sets)	Medium (8 channels, 500 Hz)	Yes (validated fear-inducing videos)	Yes (RBF SVM)	Yes (subject-dependent and -independent)	Yes (institutional approval and consent)	Moderate
Turnip et al. (2024) [16]	Yes	Yes (flight simulator with Decision Tree)	Partial (*n* = 16, mostly male, half experienced)	High (19 channels, Mitsar 202, protocol control)	Yes (landing phases rated by experts)	Yes (Fine/ Medium/ Coarse Decision Trees)	Yes (confusion matrix, per-class precision)	Yes (consent reported)	Moderate
Vempati et al. (2023) [17]	Yes	Yes (systematic review of multimodal DBs)	Yes (over 90 studies across populations)	Variable (1–128 channels discussed)	Yes (fear explicitly reported in studies)	Yes (SVM, CNN, LSTM, others)	Yes (summarized validation practices)	Yes (ethical norms reviewed)	Low

**Table 2:** Algorithms used to classify brain signals related to fear.

Algorithms	Articles	Observations
CNN, DBN, RNN, DNN	Vempati et al. (2023) [17]	Deep learning algorithms useful for complex learning
SVM	Bălan et al. (2019) [9], Ishizuka et al. (2024) [15], and Vempati et al. (2023) [17]	High accuracy in studies with clean signals
Random Forest (RF)	Bălan et al. (2019) [9] and Vempati et al. (2023) [17]	Stable performance
kNN	Bălan et al. (2019) [9] and Vempati et al. (2023) [17]	Lower accuracy, high speed
LDA	Bălan et al. (2019) [9]	Comparative linear method
SCN	Bălan et al. (2019) [9]	Good performance with feature selection
ELM + TQWT	Krishna et al. (2019) [11]	Competitive accuracy with lower computational load
EMD + DE + SVM	Ishizuka et al. (2024) [15]	Better performance than STFT and Wavelet in real time
Decision Tree	Turnip et al. (2024) [16]	92.95% accuracy in fear detection among pilots
Statistical Analysis	de Man et al. [8]	Simple detection without complex algorithms
Pattern Recognition	Alakus et al. (2020) [14]	Practical application in games (no classifier specified)

The listed studies present various approaches for classifying EEG signals to detect fear.

**Table 3:** EEG hardware used in fear detection studies and their characteristics.

Hardware	Articles	Channels	Observations
Biosemi ActiveTwo	Bălan et al. (2019) [9] and Vempati et al. (2023) [17]	32	Standard in many benchmark studies
Emotiv Epoc/Epoc+	Kim et al. (2019) [10], Alakus et al. (2020) [14], and Vempati et al. (2023) [17]	14	Portable, comfortable, widely used
Neuroscan/Mobita	Vempati et al.(2023) [17]	32	Clinical-grade equipment
Mitsar 202 Digital Amplifier	Turnip et al. (2024) [16]	19	Clinical EEG used with flight simulators
PolymatePocket MP208	Ishizuka et al. (2024) [15]	8	High resolution, portable
MyndPlay Brainband (1 sensor)	de Man et al.(2017) [8]	1	Minimalist EEG for home use
Clinical system (not specified)	Proverbio et al. (2024) [7]	NA	Used for swLORETA; no hardware details provided

**Table 4:** Databases used in fear detection studies with EEG.

Database	Articles	Type
DEAP	Bălan et al. (2019) [9], Krishna et al. (2019) [11], and Vempati et al. (2023) [17]	Induced emotions
GAMEEMO	Alakus et al. (2020) [14]	EEG from gameplay
MAHNOB, DREAMER	Vempati et al. (2023) [17]	Multimodal
Own dataset (pilot EEG)	Turnip et al. (2024) [16]	EEG in flight simulator
Custom experimental	de Man et al. (2017) [8], Proverbio et al. (2024) [7], and Ishizuka et al. (2024) [15]	EEG by design

**Table 5:** Protocols and emotional stimulation applied in EEG-based fear studies.

Article	Protocol Used	Stimulation Applied
Vempati et al. (2023) [17]	Public datasets (DEAP, MAHNOB, DREAMER)	Videos, images, and audio to induce basic emotions
Proverbio et al. (2024) [7]	Guided emotional recall	Mental evocation of fear, sadness, and joy through verbal instructions
de Man et al. (2017) [8]	Video viewing with rotating self-labeling	Fear and relaxation videos; continuous recording with potentiometer
Bălan et al. (2019) [9]	Use of DEAP database with music videos	Pre-labeled audiovisual stimuli by emotional level (valence/arousal)
Kim et al. (2019) [10]	Visualization of nighttime landscapes	Urban scenes with/without human figure to induce perceptual variations
Krishna et al.(2019) [11]	EEG with music clips (DEAP)	Emotionally preclassified music in a controlled study
Alakus et al. (2020) [14]–GAMEEMO	Emotion-labeled games with real-time EEG	Active interaction with video games (bored, relaxed, fun, fear); SAM questionnaire
Ishizuka et al. (2024) [15]	Viewing fear-inducing videos in a dark room	Selected short videos; subjective evaluation after each segment
Turnip et al. (2024) [16]	Full flight simulation with EEG	Recording during takeoff, cruising, and landing; focus on landing phase
Cao et al. (2014) [12]	Controlled emotional acting	Interpretation of basic emotions by actors; multimodal audiovisual recording
Eneddon et al. (2012) [13]	Natural emotional induction in semi-structured environment	Filmed everyday scenarios (interviews, moderated social interactions)

**Table 6:** Relevant electrodes and main findings in EEG-based fear detection studies.

Article	Relevant Electrodes	Justification / Result
Turnip et al. (2024) [16]	Fp1, T4, F3, Cz	Fp1 and T4 showed greater amplitude during fear; 92.95% accuracy using Decision Tree
Ishizuka et al. (2024) [15]	Fz, Cz, P3, Pz	Elevated activation in Fz and Cz during fear videos; 86% accuracy with EMD + SVM
Bălan et al. (2019) [9]	F3, F4, T7, T8	Frontal and temporal regions optimized emotional classification; 89.96% accuracy
Kim et al. (2019) [10]	Fz, Pz	Alpha and beta activity changes in response to nighttime visual stimuli
Krishna et al. (2019) [11]	F3, F4, Cz	Key frequency bands extracted from frontocentral zones using TQWT
Alakus et al. (2020) [14]	AF3, AF4, T7, T8	Games induced fear by activating prefrontal and temporal zones, based on SAM labels
de Man et al. (2017) [8]	Single frontal channel (approx. Fz)	Changes detected with frontal sensor during fear and relaxation videos
Proverbio et al. (2024) [7]	Left frontal areas	swLORETA identified fear origin in the left hemisphere
Vempati et al. (2023) [17]	Not specified (multi-channel)	Electrodes vary by database (DEAP, MAHNOB, etc.)
Cao et al. (2014) [12]	Not applicable	Audiovisual database (no EEG data)
Eneddon et al. (2012) [13]	Not applicable	Real interactions recorded without EEG

**Table 7:** Association between EEG frequency bands and fear-related responses.

EEG Band	Association with Fear	Articles	Observation
Gamma (>30 Hz)	High cognitive and emotional activation	Turnip et al. (2024) [16], Ishizuka et al. (2024) [15], and Bălan et al. (2019) [9]	Frequently elevated under intense fear conditions
Beta (12–30 Hz)	Conscious activation, anxiety, and stress	Turnip et al. (2024) [16], Kim et al. (2019) [10], and Bălan et al. (2019) [9]	Increases during attention-demanding tasks facing threatening stimuli
Alpha (8–13 Hz)	Reduced relaxation (decrease indicates emotional arousal)	Turnip et al. (2024) [16], Kim et al. (2019) [10], and Krishna et al. (2019) [11]	Decreases in response to negative visual or emotional stimuli
Theta (4–7 Hz)	Deep emotional processing and memory	Vempati et al. (2023) [17] and Krishna et al.(2019) [11]	Present in introspective contexts or guided emotional recall
Delta (<4 Hz)	Deep sleep, minimal relation to conscious fear	Not reported as relevant in fear-related articles	Not predominant in conscious fear states

**Table 8:** Preprocessing and processing techniques used in studies on EEG-based fear detection.

Article	Filtering and Cleaning	Processing
Turnip et al. (2024) [16]	BPF (0.5–50 Hz), low-cut 0.3 s, manual artifact removal	Flight phase segmentation, band extraction (*α*, *β*1, *β*2, *γ*), epoch averaging
Ishizuka et al. (2024) [15]	EMD decomposition, 5 s window segmentation	Mean and differential entropy of IMFs, classification with SVM
Bălan et al. (2019) [9]	DEAP preprocessing, bandpass filters, normalization	PSD, STFT, PCA reduction, time-frequency analysis
Kim et al. (2019) [10]	Bandpass filter (alpha/beta), visual inspection for artifacts	Relative band power, topographic analysis
Krishna et al. (2019) [11]	Normalization, multiband TQWT	Statistical features per sub-band, classification with ELM
Alakus et al. (2020) [14]	Sinc filter (5th order), motion artifact removal	Signal averaging per session, emotional labeling (SAM)
de Man et al. (2017) [8]	No advanced filtering specified	Single-channel amplitude analysis, continuous self-labeling
Proverbio et al. (2024) [7]	Manual artifact rejection, ERP averaging, channel interpolation	Brain source analysis using swLORETA
Vempati et al. (2023) [17]	Filtering and normalization based on dataset used	Combination of statistics, spectra, and deep learning
Cao et al. (2014) [12]	Not applicable (audiovisual dataset)	Not applicable
Eneddon et al. (2012) [13]	Not applicable (audiovisual recordings)	Not applicable

**Table 9:** Emotion models used in EEG-based fear detection studies.

Emotion Model	Brief Description	Articles Using It	Key Observations
Ekman (basic emotions)	Discrete model identifying 6 universal emotions: fear, joy, sadness, anger, surprise, and disgust	Proverbio et al. (2024) [7], de Man et al. (2017) [8], and Ishizuka et al. (2024) [15]	Useful for direct classification tasks, especially fear
Russell’s Circumplex (Valence–Arousal)	Dimensional model with two axes: valence (pleasant/unpleasant) and arousal (intensity)	Bălan et al. (2019) [9], Krishna et al. (2019) [11], Alakus et al. (2020) [14], and Vempati et al. (2023) [17]	Widely used with tools like SAM; allows for representation of complex emotions
SAM (Self-Assessment Manikin)	Graphic tool for self-assessment of emotions in terms of valence and arousal	Alakus et al. (2020) [14] and Vempati et al. (2023) [17]	Applied as a subjective scale for emotion labeling
5 Levels of Fear	Specific model categorizing fear as: none, low, moderate, significant, and extreme	Turnip et al. (2024) [16]	Adapted to flight simulation; useful for fine-grained fear classification
Guided Acting (posed emotions)	Emotions portrayed by actors following specific instructions (performance-based model)	Cao et al. (2014) [12]	Not a formal theoretical model; relies on scripted expressions of basic emotions
Naturalistic Emotion Induction	Experimental model based on real, spontaneous stimuli	Eneddon et al. (2012) [13]	Seeks to capture genuine emotional responses without artificial instructions

**Table 10:** Stimuli and experiment duration in studies related to EEG-based fear detection.

Article	Experiment Duration	Type of Stimulus
Turnip et al. (2024) [16]	Approximately 30 min (complete flight simulation)	Flight simulator (critical landing phase)
Ishizuka et al. (2024) [15]	3 videos of 90 s each	Short videos designed to induce fear
Bălan et al. (2019) [9]	1 min per video	Music videos (DEAP dataset)
Kim et al. (2019) [10]	Not precisely specified; inferred to be less than 10 min	Urban nighttime images
Krishna et al. (2019) [11]	1 min per clip	Emotional music clips (DEAP)
Alakus et al. (2020) [14]	5 min per game session	Emotionally charged video games (GAMEEMO)
de Man et al. (2017) [8]	Fear and relaxation videos (4 min)	Continuous viewing with self-reported emotional labeling
Proverbio et al. (2024) [7]	Guided emotional recall (6 min)	Verbally induced mental recollection
Vempati et al. (2023) [17]	Varies by dataset (1 min)	DEAP, DREAMER, MAHNOB-HCI (multimodal)
Cao et al. (2014) [12]	Recorded phrases (1 to 3 s)	Voice performances and emotional expression
Eneddon et al. (2012) [13]	Real-life situations, variable duration	Recorded interviews and natural tasks

**Table 11:** Population sampling characteristics in the included studies.

Author(s) and Year	Sampling Description
Proverbio et al. (2024) [7]	Partial (*n* = 20, university students)
de Man et al. (2017) [8]	Partial (*n* = 30, university students and staff)
Bălan et al. (2020) [9]	Yes (DEAP dataset: 32 balanced participants)
Kim et al. (2019) [10]	Partial (*n* = 30, limited age and demographic criteria)
Krishna et al. (2020) [11]	Not reported
Cao et al. (2014) [12]	Yes (91 actors, varied in age, ethnicity, and gender)
Sneddon et al. (2012) [13]	Yes (20 participants with age and cultural diversity)
Alakus et al. (2020) [14]	Partial (28 young participants, no detailed demographic report)
Ishizuka et al. (2024) [15]	Limited (*n* = 6, usable EEG in 5, all young)
Turnip et al. (2024) [16]	Partial (16 participants, 15 male; half with simulator experience)
Vempati et al. (2023) [17]	Not reported

**Table 12:** Hybrid EEG-based systems for fear detection.

Author and Year	Hybrid System	Main Findings	Limitations
Proverbio et al. (2024) [7]	EEG (128-ch) + verbally guided emotional recall + pictograms + source reconstruction (sLORETA)	Discrete fear, joy, and sadness states showed distinct cortical activations; fear linked to right STG and insula.	Dependent on subjective imagination; only healthy participants; no peripheral or real-time validation.
de Man et al. (2017) [8]	Single-sensor EEG (MyndPlay) + rotary potentiometer + post-stimulus questionnaire	High-beta and low-gamma bands correlated with fear peaks; single-channel EEG successfully captured fear-related signals.	Limited spatial resolution; response timing variability; lab-controlled environment with audiovisual stimuli only.
Bălan et al. (2019) [9]	EEG (DEAP dataset) + peripheral signals (GSR, EMG, respiration, temperature, PPG)	High classification accuracy for fear using fractal and entropy-based EEG features combined with physiological data; DNN and SVM among best performers.	Relies on pre-recorded emotional labels; no real-time validation; performance varies across classifiers and emotional scales.
Kim et al. (2019) [10]	Mobile EEG (Emotiv EPOC) + nightscape images + fear self-report survey	Fear higher in urban settings without adult presence; alpha/beta wave changes aligned with perceived fear.	Static images only; low sample diversity; no ecological immersion.
Krishna et al. (2019) [11]	24-ch EEG (frontal bipolar) + audiovisual stimuli + SAM questionnaire + TQWT + ELM classifier	87.1% classification accuracy; 86.5% for fear using FP2–F4; outperformed ANN/SVM in same dataset.	Small homogeneous sample; no immersive or real-time testing; fixed-channel evaluation only.
Ishizuka et al. (2024) [15]	8-ch EEG (PolymatePocket MP208) + horror video clips + EMD (mean, DE) + SVM	EMD outperformed STFT and DWT; 87.7% (subject-dependent), 80.3% (independent); demonstrated real-time potential.	Small sample (5); post hoc labeling; manual interview-based tagging; no deep learning.
Turnip et al. (2024) [16]	EEG (19-ch Mitsar 202) + flight simulation + amplitude of *α*, *β*1, *β*2, *γ* + Decision Tree	Classified fear in 5 levels with 92.95% accuracy during simulated landings; supports pilot assessment.	Small sample; no real-flight test; no deep learning; EEG-only modality.

## Data Availability

No new data were created or analyzed in this study.

## Supplementary Materials

The following supporting information can be downloaded at: https://www.mdpi.com/article/10.3390/brainsci15080815/s1, File S1: PRISMA 2020 Checklist. Reference [6] is cited in the supplementary materials.

## Abbreviations

The following abbreviations are used in this manuscript:

EEG	Electroencephalography
AI	Artificial Intelligence
SVM	Support Vector Machine
CNN	Convolutional Neural Network
DWT	Discrete Wavelet Transform
EMD	Empirical Mode Decomposition
ICA	Independent Component Analysis
SAM	Self-Assessment Manikin
VR	Virtual Reality
Fp1, F3, Fz, T4, T7, T8	Common EEG Electrode Locations
DEAP	Database for Emotion Analysis using Physiological Signals
MAHNOB	MAHNOB-HCI Multimodal Database
DBN	Deep Belief Network
SECIHTI	Secretariat of Science, Humanities, Technology and Innovation
