# Supplementary material for: Fear Detection Using Electroencephalogram and Artificial Intelligence: A Systematic Review
Source: Brain Sci. 2025 Jul 29;15(8):815. doi: 10.3390/brainsci15080815 (PMC12384336; doi:10.3390/brainsci15080815)
Supplement: Supplementary file 1 [file brainsci-15-00815-s001.zip › brainsci-3729736-supplementary.pdf]

PRISMA 2020 Checklist

Section and Topic	Item #	Checklist item	Location where item is reported
**TITLE**			
Title	1	Identify the report as a systematic review.	Title page
**ABSTRACT**			
Abstract	2	See the PRISMA 2020 for Abstracts checklist.	Structured Abstract
**INTRODUCTION**			
Rationale	3	Describe the rationale for the review in the context of existing knowledge.	Introduction, paragraph 1
Objectives	4	Provide an explicit statement of the objective(s) or question(s) the review addresses.	Introduction, last paragraph
**METHODS**			
Eligibility criteria	5	Specify the inclusion and exclusion criteria for the review and how studies were grouped for the syntheses.	Methods - Section 1.3 Inclusion and exclusion Criteria
Information sources	6	Specify all databases, registers, websites, organisations, reference lists and other sources searched or consulted to identify studies. Specify the date when each source was last searched or consulted.	Methods - Section 1.2 Search Strategy
Search strategy	7	Present the full search strategies for all databases, registers and websites, including any filters and limits used.	Methods - Section 1.2 Search Strategy
Selection process	8	Specify the methods used to decide whether a study met the inclusion criteria of the review, including how many reviewers screened each record and each report retrieved, whether they worked independently, and if applicable, details of automation tools used in the process.	Methods - Section 1.3 Study Selection 1.6 Quality Assessment and Risk of Bias
Data collection process	9	Specify the methods used to collect data from reports, including how many reviewers collected data from each report, whether they worked independently, any processes for obtaining or confirming data from study investigators, and if applicable, details of automation tools used in the process.	Methods - Section 2.4 Data Extraction
Data items	10a	List and define all outcomes for which data were sought. Specify whether all results that were compatible with each outcome domain in each study were sought (e.g. for all measures, time points, analyses), and if not, the methods used to decide which results to collect.	Methods - Section 1.5 Data Extraction and Synthesis
	10b	List and define all other variables for which data were sought (e.g. participant and intervention characteristics, funding sources). Describe any assumptions made about any missing or unclear information.	Methods - Section 1.5 Data

PRISMA 2020 Checklist

Section and Topic	Item #	Checklist item	Location where item is reported
			Extraction and Synthesis
Study risk of bias assessment	11	Specify the methods used to assess risk of bias in the included studies, including details of the tool(s) used, how many reviewers assessed each study and whether they worked independently, and if applicable, details of automation tools used in the process.	Methods – Section 1.6 Risk of Bias Assessment
Effect measures	12	Specify for each outcome the effect measure(s) (e.g. risk ratio, mean difference) used in the synthesis or presentation of results.	Not applicable (no effect size estimation)
Synthesis methods	13a	Describe the processes used to decide which studies were eligible for each synthesis (e.g. tabulating the study intervention characteristics and comparing against the planned groups for each synthesis (item #5)).	Methods - Section 1.1 and 1.3
	13b	Describe any methods required to prepare the data for presentation or synthesis, such as handling of missing summary statistics, or data conversions.	Methods - Section 1.5 and Results
	13c	Describe any methods used to tabulate or visually display results of individual studies and syntheses.	Results - Tables 1 to 9 and Figure 1
	13d	Describe any methods used to synthesize results and provide a rationale for the choice(s). If meta-analysis was performed, describe the model(s), method(s) to identify the presence and extent of statistical heterogeneity, and software package(s) used.	A meta-analysis was not performed due to the methodological heterogeneity across studies in terms of EEG acquisition settings, classification algorithms, stimulation protocols, and outcome measures. Therefore, a narrative and comparative synthesis was conducted instead.
	13e	Describe any methods used to explore possible causes of heterogeneity among study results (e.g. subgroup analysis, meta-regression).	Not applicable. No meta-analysis was conducted; therefore, no exploration of statistical heterogeneity was performed.
	13f	Describe any sensitivity analyses conducted to assess robustness of the synthesized results.	Not applicable. No quantitative synthesis or sensitivity analyses were conducted.
Reporting bias assessment	14	Describe any methods used to assess risk of bias due to missing results in a synthesis (arising from reporting biases).	Methods - Section 1.6 and Results - Table 5
Certainty assessment	15	Describe any methods used to assess certainty (or confidence) in the body of evidence for an outcome.	A formal certainty assessment method such as GRADE was not applied. However, the quality and methodological rigor of the included studies were

PRISMA

PRISMA 2020 Checklist

Section and Topic	Item #	Checklist item	Location where item is reported
			qualitatively discussed in the risk of bias assessment and in the Discussion section.
**RESULTS**			
Study selection	16a	Describe the results of the search and selection process, from the number of records identified in the search to the number of studies included in the review, ideally using a flow diagram.	Methods - Section Figure 1 and Results - Section 1.1
	16b	Cite studies that might appear to meet the inclusion criteria, but which were excluded, and explain why they were excluded.	No specific table of excluded studies was included. However, during the screening process, several articles were excluded due to not fulfilling key criteria (e.g., absence of EEG data, unrelated emotion focus, or non-human subjects).
Study characteristics	17	Cite each included study and present its characteristics.	Results - Tables 1 to 9
Risk of bias in studies	18	Present assessments of risk of bias for each included study.	Results - Table 5
Results of individual studies	19	For all outcomes, present, for each study: (a) summary statistics for each group (where appropriate) and (b) an effect estimate and its precision (e.g. confidence/credible interval), ideally using structured tables or plots.	Structured tables present summary performance metrics (e.g., accuracy (Results – tables 5 and 6)) for each study. However, effect estimates and confidence intervals were not reported, as the primary studies used heterogeneous metrics and designs.
Results of syntheses	20a	For each synthesis, briefly summarise the characteristics and risk of bias among contributing studies.	Results – Tables 1 – 9 Studies included in each synthesis were summarized in comparative tables. Key characteristics such as EEG configuration, stimulation type, and AI models were extracted. Risk of bias was qualitatively assessed.
	20b	Present results of all statistical syntheses conducted. If meta-analysis was done, present for each the summary estimate and its precision (e.g. confidence/credible interval) and measures of statistical heterogeneity. If comparing groups, describe the direction of the effect.	Not applicable. No statistical synthesis or meta-analysis was conducted due to the methodological and metric

Section and Topic	Item #	Checklist item	Location where item is reported
			heterogeneity of the included studies.
	20c	Present results of all investigations of possible causes of heterogeneity among study results.	Not applicable
	20d	Present results of all sensitivity analyses conducted to assess the robustness of the synthesized results.	Not applicable. No investigations of heterogeneity were conducted, as no statistical synthesis
Reporting biases	21	Present assessments of risk of bias due to missing results (arising from reporting biases) for each synthesis assessed.	No formal analysis of reporting bias was conducted. However, potential publication bias is acknowledged due to the limited number of studies available and the heterogeneity in reporting formats. This was qualitatively considered in the discussion.
Certainty of evidence	22	Present assessments of certainty (or confidence) in the body of evidence for each outcome assessed.	No formal assessment tool such as GRADE was used. However, the overall quality and consistency of the evidence were discussed qualitatively based on methodological rigor, and data reporting across studies.
**DISCUSSION**			
Discussion	23a	Provide a general interpretation of the results in the context of other evidence.	The results were interpreted in light of prior research on EEG and AI for fear detection. Each subsection includes a contextual discussion, allowing for area-specific interpretation of findings and alignment with existing evidence.
	23b	Discuss any limitations of the evidence included in the review.	The included studies showed considerable methodological variability, limited sample sizes, and inconsistent reporting standards. These limitations reduce comparability and affect the overall strength of the evidence synthesized. These concerns are addressed through focused discussions included in

Section and Topic	Item #	Checklist item	Location where item is reported
			each subsection of the results.
	23c	Discuss any limitations of the review processes used.	This review used three major databases (Scopus, Clarivate, and PubMed), and the selection and evaluation processes included peer review and triangulated author coding to enhance methodological rigor. However, the protocol was not preregistered, and the synthesis remained narrative due to study heterogeneity.
	23d	Discuss implications of the results for practice, policy, and future research.	The findings suggest potential applications in clinical settings, elderly care, and emotional monitoring through AI-enhanced EEG systems. However, the review identified critical gaps, such as the lack of standardized stimulation protocols, limited sample sizes, and poor cross-validation practices. Ethical concerns regarding the manipulation of emotional states in vulnerable populations were also noted, highlighting the need for strict ethical frameworks in future research. These limitations must be addressed to strengthen the practical and scientific validity of fear detection systems.
**OTHER INFORMATION**			
Registration and protocol	24a	Provide registration information for the review, including register name and registration number, or state that the review was not registered.	The review was not registered in any public registry.
	24b	Indicate where the review protocol can be accessed, or state that a protocol was not prepared.	No review protocol was prepared or made publicly available.
	24c	Describe and explain any amendments to information provided at registration or in the protocol.	Not applicable
Support	25	Describe sources of financial or non-financial support for the review, and the role of the funders or sponsors in the review.	Funding statement -

PRISMA 2020 Checklist

Section and Topic	Item #	Checklist item	Location where item is reported
			Acknowledgments section
Competing interests	26	Declare any competing interests of review authors.	Conflict of Interest section
Availability of data, code and other materials	27	Report which of the following are publicly available and where they can be found: template data collection forms; data extracted from included studies; data used for all analyses; analytic code; any other materials used in the review.	No supplementary materials (e.g., data extraction forms, analytic code, or raw datasets) have been made publicly available. Relevant synthesis tables are included within the manuscript.

Page, M.J.; McKenzie, J.E.; Bossuyt, P.M.; Boutron, I.; Hoffmann, T.C.; Mulrow, C.D.; Shamseer, L.; Tetzlaff, J.M.; Akl, E.A.; Brennan, S.E.; et al. Declaración PRISMA 2020: Una Guía Actualizada para la Publicación de Revisiones Sistemáticas. *Rev. Esp. Cardiol.* **2021**, *74*, 790–799. https://doi.org/10.1016/j.recesp.2021.06.016.
